# Supplementary material for: Dietary Flavonoid Intake and Chronic Sensory Conditions: A Scoping Review
Source: Antioxidants (Basel). 2022 Jun 21;11(7):1214. doi: 10.3390/antiox11071214 (PMC9311508; doi:10.3390/antiox11071214)
Supplement: Supplementary file 1 [file antioxidants-11-01214-s001.zip › antioxidants-1737242-supplementary.pdf]

Supplementary Table S1. Quality assessment of case-control studies.

Criteria[26]	Ma et al. (2015) [10]
Were the groups comparable other than the presence of disease in cases or the absence of disease in controls?	Yes
Were cases and controls matched appropriately?	No
Were the same criteria used for identification of cases and controls?	No
Was exposure measured in a standard, valid and reliable way?	Yes
Was exposure measured in the same way for cases and controls?	Yes
Were confounding factors identified?	Yes
Were strategies to deal with confounding factors stated?	Yes
Were outcomes assessed in a standard, valid and reliable way for cases and controls?	Yes
Was the exposure period of interest long enough to be meaningful?	Unclear
Was appropriate statistical analysis used?	Yes

Supplementary Table S2. Quality assessment of cross-sectional studies.

Criteria[26]	Mahoney & Loprinzi. 2014 [15]	Kim et al. 2018 [27]
Were the criteria for inclusion in the sample clearly defined?	Yes	Yes
Were the study subjects and the setting described in detail?	Yes	Yes
Was the exposure measured in a valid and reliable way?	Yes	No
Were objective, standard criteria used for measurement of the condition?	Yes	Yes
Were confounding factors identified?	Yes	Yes
Were strategies to deal with confounding factors stated?	Yes	Yes
Were the outcomes measured in a valid and reliable way?	Yes	Yes
Was appropriate statistical analysis used?	Yes	Yes

Supplementary Table S3. Quality assessment of cohort studies.

Criteria[26]	Ramdas et al. 2012 [13]	Gopinath et al. 2018 [6]	Kang et al. 2018 [12]	Gopinath et al. 2020 [28]	Deteram et al. 2021 [30]	Tang et al. 2021 [29]
Were the two groups similar and recruited from the same population?	Yes	Yes	Yes	Yes	Yes	Yes
Were the exposures measured similarly to assign people to both exposed and unexposed groups?	Yes	Yes	Yes	Yes	Yes	Yes
Was the exposure measured in a valid and reliable way?	Yes	Yes	Yes	Yes	Yes	Yes
Were confounding factors identified?	Yes	Yes	Yes	Yes	Yes	Yes
Were strategies to deal with confounding factors stated?	Yes	Yes	Yes	Yes	Yes	Yes
Were the groups/participants free of the outcome at the start of the study (or at the moment of exposure)?	Yes	Yes	Yes	Yes	No	Yes
Were the outcomes measured in a valid and reliable way?	Yes	Yes	Yes	Yes	Yes	Yes
Was the follow up time reported and sufficient to be long enough for outcomes to occur?	Yes	Yes	Yes	Yes	Yes	Yes
Was follow up complete, and if not, were the reasons to loss to follow up described and explored?	No, yes	Unclear, unclear	Unclear, unclear	Unclear, no	No, no	Unclear, no
Were strategies to address incomplete follow up utilized?	Unclear	Unclear	Unclear	Unclear	Unclear	Unclear
Was appropriate statistical analysis used?	Yes	Yes	Yes	Yes	Yes	Yes

Supplementary Table S4. Quality assessment of randomised controlled trials.

Criteria[26]	Terai et al. (2014) [11]
Was true randomization used for assignment of participants to treatment groups?	Yes
Was allocation to treatment groups concealed?	Yes
Were treatment groups similar at the baseline?	Yes
Were participants blind to treatment assignment?	No
Were those delivering treatment blind to treatment assignment?	Yes
Were outcomes assessors blind to treatment assignment?	Yes
Were treatment groups treated identically other than the intervention of interest?	Yes
Was follow up complete and if not, were differences between groups in terms of their follow up adequately described and analyzed?	Yes
Were participants analyzed in the groups to which they were randomized?	Yes
Were outcomes measured in the same way for treatment groups?	Yes
Were outcomes measured in a reliable way?	Yes
Was appropriate statistical analysis used?	Yes
Was the trial design appropriate, and any deviations from the standard RCT design (individual randomization, parallel groups) accounted for in the conduct and analysis of the trial?	Yes

Supplementary Figure S1.Screen capture of the search strategy utilised in OVID. This search was re-run on 14/06/2022 as a copy of the original search was not screen captured. Note that there was no year limit in the original search, but to reflect the original search done in 2021, we have restricted abstracts up to 2021.

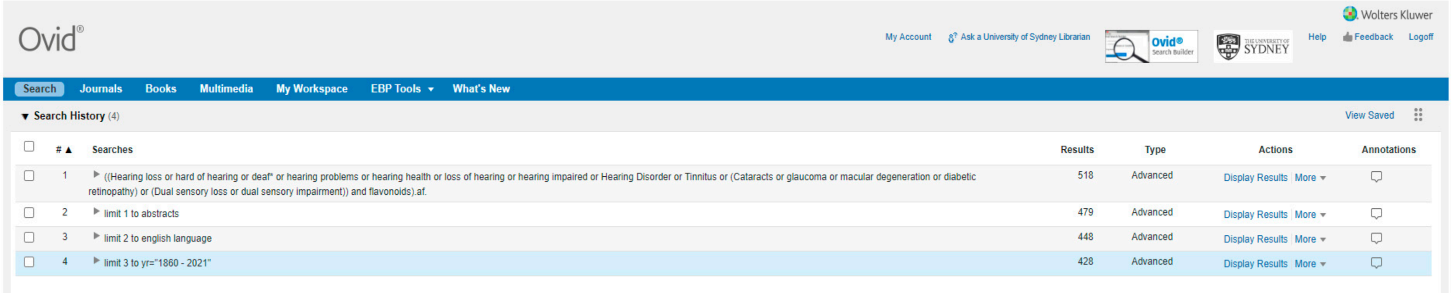

Search History (4)

#	Searches	Results	Type	Actions	Annotations
1	((Hearing loss or hard of hearing or deaf* or hearing problems or hearing health or loss of hearing or hearing impaired or Hearing Disorder or Tinnitus or (Cataracts or glaucoma or macular degeneration or diabetic retinopathy) or (Dual sensory loss or dual sensory impairment)) and flavonoids).af.	518	Advanced	Display Results More	
2	limit 1 to abstracts	479	Advanced	Display Results More	
3	limit 2 to english language	448	Advanced	Display Results More	
4	limit 3 to yr="1860 - 2021"	428	Advanced	Display Results More	
